# Dentoalveolar and Soft Tissue Changes Following en-Masse Anterior Retraction With Different Force Vectors in Subjects With Bidental Protrusion: A Retrospective Evaluation

**DOI:** 10.7759/cureus.45274

**Published:** 2023-09-14

**Authors:** Prem Vishva, Ravindra Kumar Jain

**Affiliations:** 1 Orthodontics and Dentofacial Orthopedics, Saveetha Dental College and Hospitals, Saveetha Institute of Medical and Technical Sciences, Saveetha University, Chennai, IND; 2 Dentistry, Saveetha Dental College and Hospitals, Saveetha Institute of Medical and Technical Sciences, Saveetha University, Chennai, IND

**Keywords:** tads, transpalatal arch, retraction, skeletal anchorage, force vector

## Abstract

Introduction

The aim of the study is to determine the effects of different vertical force vectors acting on dentoalveolar and soft tissues while applying a retraction force using various anchorage sources and their effects on these tissues.

Material and methods

Based on the selection criteria, a total of 35 patient case records with Angle's Class I bidental malocclusion and incompetent lips treated with four premolar extractions were included. Retraction was achieved using a NiTi coil spring with two different force vectors. The anchorage in group 1 was enhanced by a transpalatal arch (TPA) and Lower Stabilizing Arch (LSA) in the upper and lower arch, respectively, with a force vector parallel to the occlusal plane, whereas in group 2, the force vector using Temporary Anchorage Devices (TADs) was 15 - 20 degrees to the occlusal plane. The skeletal, dental, and soft tissue were obtained using 40 parameters. Intra-group comparisons between pre- and post-treatment records were conducted using a paired t-test, while inter-group comparisons were conducted using an independent t-test.

Result

Significant anchor loss was observed in group 1, indicated by mesial movement of molar crowns by (-2.10±0.50) in the maxillary arch and (-1.75±0.38) in the mandibular arch. Distal movement of incisors following premolar extractions with both studied force vectors resulted in an improvement in lip procumbency and incisor inclinations, without any significant skeletal changes. Molar mesial movement was observed in subjects treated with conventional anchorage.

Conclusion

En masse anterior retraction did lead to anchorage loss when carried out without TADs. Altering the force vector did not produce significant changes in tooth movement along the vertical plane.

## Introduction

Patients with dentoalveolar bidental protrusion frequently exhibit heightened incisor inclination and lip protrusion, resulting in muscular imbalance and lip incompetence [1,2]. Attaining proper lip position and shape is a prerequisite for achieving favorable facial aesthetics. Essential criteria for predicting changes in facial profile after orthodontic treatment include the extent of anterior tooth retraction and lip movement [3]. Enhancing the soft-tissue profile depends on several facial anatomical variables, including lip thickness, facial muscle activity, and ethnicity [4,5]. Retracting incisors in cases of bidental protrusion leads to decreased inclinations and improved soft tissues, as reported in studies [6,7].

In orthodontic treatment for cases of bidental proclination, premolar teeth extraction is performed to create space for anterior tooth retraction and achieve the necessary soft tissue changes [8]. Patients requiring maximum anchorage reinforcement with temporary anchorage devices (TADs) should consider them, as they offer greater reliability and efficiency [9]. Conventional en masse retraction in patients with a vertical growth pattern and gummy smile may worsen the bite due to extrusion of the upper molar teeth. The utilization of appropriate mechanics and mini screws can lead to bodily translation and incisor root movement. By adjusting the heights of power arms during anterior teeth retraction, the inclinations of anterior teeth can be controlled [10]. A Finite Element Method (FEM) study conducted by Chetan et al. examined the impact of altering the vertical position of mini-implants on the position of anterior teeth. The study concluded that changing the implant height can influence the degree of anterior teeth intrusion [11].

Very few clinical studies have been reported in the past on the impact of different vertical force vectors on the position of anterior teeth and the related soft tissue changes during retraction. Original with correction: Hence, in the current investigation, an effort was undertaken to determine how different vertical force vectors acting on dentoalveolar and soft tissues while applying a retraction force utilizing various anchoring sources affected those tissues. Correction: Thus, the current investigation aims to determine the influence of different vertical force vectors on dentoalveolar and soft tissues during retraction, utilizing various anchoring sources.

## Materials and methods

The present study was retrospective in design and was conducted using the mid-treatment records of patients who underwent orthodontic treatment with extraction of four premolars at the Department of Orthodontics, Saveetha Dental College and Hospitals, Chennai, India, from January 2018 to January 2021. Case records of 245 cases treated with all first premolar extraction were selected based on the eligibility criteria from the institutional case record archives.

Inclusion criteria

Case records of patients with class I malocclusion and bimaxillary protrusion in the age group of 15-35 years treated with 022 MBT metal brackets and Group A anchorage (Maximum anchorage) for en-masse retraction of the anterior segment using NiTi (Nickel Titanium) coil springs with conventional dental or TADs assisted anchorage. 

Exclusion criteria

Case records of subjects treated with friction mechanics for en-masse retraction, poor-quality photographs, and lateral cephalograms. Records of patients, in whom en-masse retraction was done with E-chains. Patients with missing upper or lower first molars with periodontal compromised dentition.

Methodology

Based on the criteria, a total of 35 case records satisfying the inclusion criteria were isolated. They were divided into two groups: Group I: Anchorage reinforcement with transpalatal arch (TPA) and Lingual arch; Group II: Anchorage reinforcement with upper and lower inter-radicular mini-screws placed between the first molar and second premolar teeth. 

**Figure 1:**
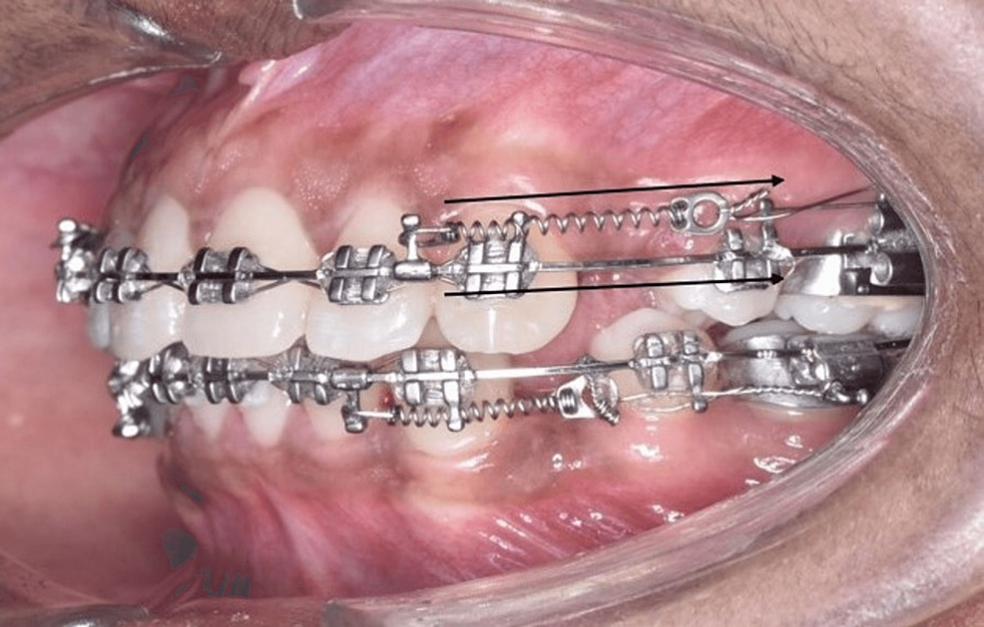
Retraction of anterior teeth with a force vector parallel to the occlusal plane in group 1

In group I, 15 case records of patients were analyzed and included in the study. In all group 1 patients, anchorage reinforcement was achieved using a soldered TPA and a lingual stabilizing arch cemented to the upper and lower first molars, respectively, prior to anterior teeth retraction. In all patients in group 1, intra-oral photographs and case records revealed that retraction was done with a 9mm closed heavy Niti Coil Spring (G&H Orthodontics®). The retraction for all the patients was done on a 0.019 x 0.025 inch reverse curve stainless steel archwire, that had soldered hooks between the brackets of the maxillary lateral incisor and canine teeth. Intra-oral image of the patient was used to select cases with the force vectors aligned parallel to the occlusal plane as shown in Figure 1.

**Figure 2:**
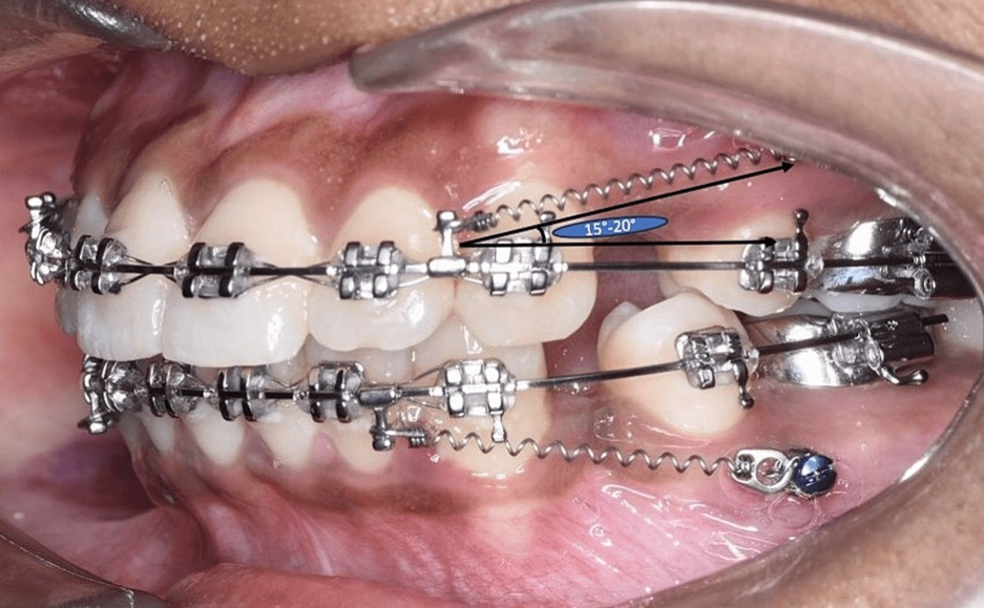
Group 2 mini-implant-aided anchorage reinforcement

In group 2, 20 patient case records were included in which, Titanium mini-implants (FavAnchor® Microimplants color code: pink) were utilized for anchorage reinforcement. The records revealed that the Mini implants (1.6mm in diameter and 8mm in length) were inserted in the junction of attached gingiva and movable mucosa between the first molar and second bicuspid at angulations of 60° to 90° to the buccal surface of the alveolus. Based on the inclusion criteria a 9mm closed heavy NiTi coil spring, weighing 250g, was applied to the group using a 0.010" stainless steel ligature wire. The records showed that in all patients, the retraction was done on 0.019x0.025 inch stainless steel archwire with posted hooks between the brackets of the maxillary lateral incisors and canines. Intra-oral image of the patient was used to select cases with force vectors approximately 15°-20° to the occlusal plane, as shown in Figure 2.

All pre-treatment (T0) and post-treatment (T1) digital lateral cephalograms were analyzed using full version Facad® Orthodontic tracing software. Table 1 gives a full description of all the parameters assessed in this study. Two new reference planes were used for calculating the amount of horizontal and vertical tooth movement. The horizontal reference plane (HRP) was set at the Sella and oriented 7° below the sella-nasion line. The vertical reference plane (VRP) was positioned perpendicular to the HRP plane.

**Table 1:** Description of all the cephalometric parameters assessed in this study 1: S (Sella), 2: N (Nasion), 3:po (Porion), 4:or (Orbitale), 5:go (Gonion), 6:me (Menton), 7:pog (Pogonion ), 8: Pn (Soft tissue Nose Tip), 9:pog' (Soft tissue Pogonion), 10:Sm (Sub Mentale), 11:A (A point convexity of maxilla), 12:B (B point in convexity of mandible), 13:Upper 1 (Upper incisor), 14:U1 Apex (Upper 1 Root), 15:U1 Center (Upper 1 center), 16: U1 Edge (Upper 1 Crown tip), 17:L1 Apex (Lower 1 Root), 18:L1 Center (Lower 1 center), 19:L1 Edge (Lower 1 crown tip), 20:U6 (Upper molar crown) 21:U6 (Upper molar Root), 22:L6 (Lower molar crown) 23:L6 (Lower molar root), 24:Gla (Glabella), 25:N' (Soft tissue nasion), 26: Sn (Subnasale), 27: SLs (Labialis superior), 28: Sto (Stomion), 29: Li (Labial Inferior), 30:SLi (Superior Lower labial) 31:HL (H line), 33:HRP (Horizontal Reference Plane), 34:VRP (Vertical Reference Plane), 35: HL (H line drawing tangent to chin and upper lip), 36:FH (Line from Porion to Orbital), 37:SN (line connecting Sella and Nasion), 38:NA (line connecting Nasion and A point), 39:NB (line connecting Nasion and A point), 40:IMPA (Lower incisor mandibular plane angle)

Skeletal	Dental		Soft tissue	
SNA (82 ±2°) Angle between the line SN and NA	Upper 1 - SN (102 ± 2°) Angle between Long axis of Upper Incisor to SN plane		Facial angle (90 ± 2°) FH plane - N' pog’ line	
SNB (80±2°) Angle between the line SN and NB	IMPA - (90 ± 3°) Angle between Long axis of Lower Incisor to mandibular plane		Ls Curvature (2-5mm) Line drawn perpendicular to FH plane tangent to upper lip	
ANB (0 ± 2°) Difference between SNA and SNB	Interincisal Angle (131 ± 4°) Angle between Long axis of Upper and lower incisor		A' to N' pog' (2± 2mm) Skeletal convexity	
	HRP - Lower incisor Center Extrusion / intrusion		HL angle (7 ± 15°) H line (chin to upper lip) - N' - pog’	
	HRP - L1 Apex Extrusion / intrusion		PRN to HL (12mm) Pn - H line Nose prominence	
	HRP - L Edge Extrusion / intrusion		SLs - HL (5mm) The upper sulcus depth is measured from the H-line.	
	HRP - U1 Apex Extrusion / intrusion		A - Sn (15mm) Upper lip thickness	
	HRP - U1 Center Extrusion / intrusion		Ls Strain (12mm) Horizontally from maxillary central incisor to the vermilion border of the upper lip.	
	HRP - U1 Edge Extrusion / intrusion		Strain factor Upper lip strain minus upper lip thickness	
	VRP - L1 Apex Tipping / translation		Li - HL (0mm) Lips are front to H line	
	VRP - L1 Center Tipping / translation		SLi - HL (5mm) Lower sulcus depth	
	VRP - L1 Edge Tipping / translation		Chin thickness (10-12mm) Pog - pog’	
	VRP - U1 Apex Tipping / translation		Nasolabial angle (90° - 110°)	
	VRP - U1 Center Tipping / translation		Mentolabial sulcus	
	VRP - U1 Edge Tipping / translation			
	VRP-U6 Molar Crown Tipping / translation			
	VRP-U6 Molar Root Tipping / translation			
	VRP-L6 Molar Crown Tipping / translation			
	VRP-L6 Molar Root Tipping / translation			
	HRP-U6 Molar Crown Extrusion / intrusion			
	HRP-U6 Molar Root Extrusion / intrusion			
	HRP-L6 Molar Crown Extrusion / intrusion			
	HRP-L Molar Root Extrusion / intrusion			

**Figure 3:**
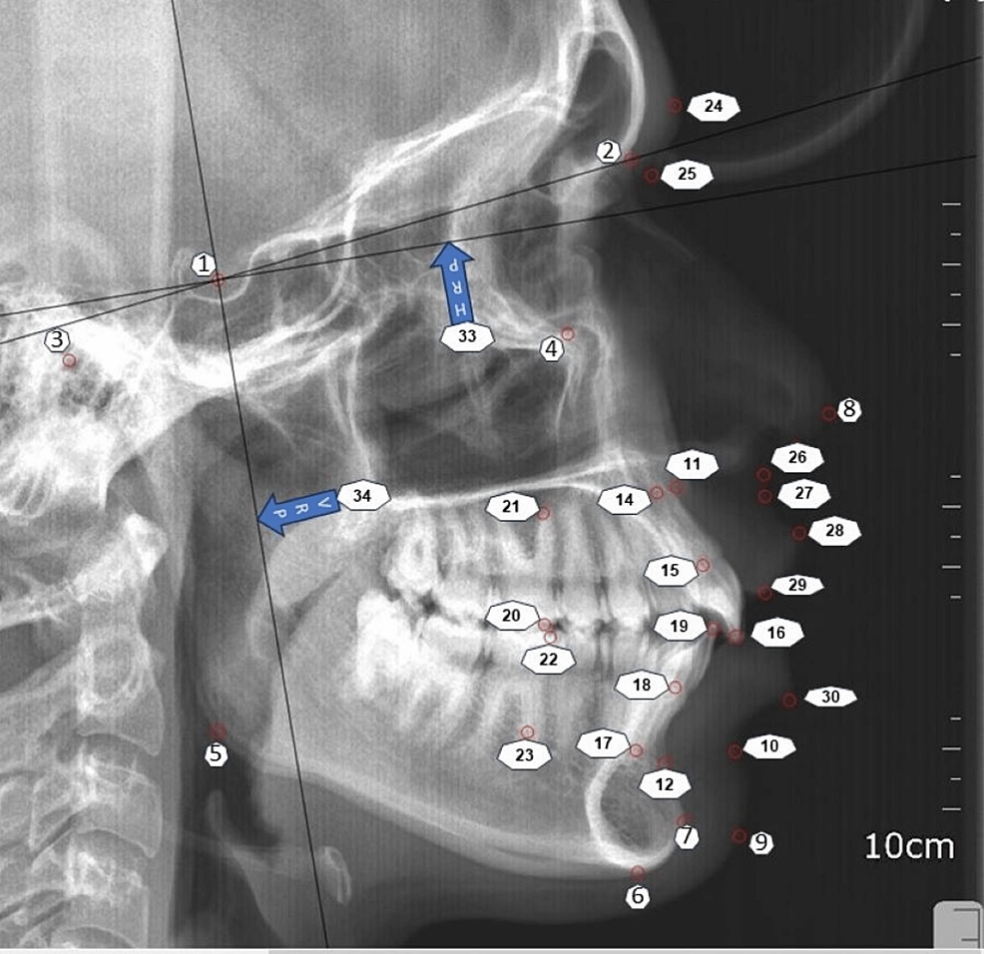
All the cephalometric parameter points assessed in this study Description of all the cephalometric parameters assessed in this study 1: S (Sella), 2: N (Nasion), 3:po (Porion), 4:or (Orbitale), 5:go (Gonion), 6:me (Menton), 7:pog (Pogonion ), 8: Pn (Soft tissue Nose Tip), 9:pog' (Soft tissue Pogonion), 10:Sm (Sub Mentale), 11:A (A point convexity of maxilla), 12:B (B point in convexity of mandible), 13:Upper 1 (Upper incisor), 14:U1 Apex (Upper 1 Root), 15:U1 Center (Upper 1 center), 16: U1 Edge (Upper 1 Crown tip), 17:L1 Apex (Lower 1 Root), 18:L1 Center (Lower 1 center), 19:L1 Edge (Lower 1 crown tip), 20:U6 (Upper molar crown)  21:U6 (Upper molar Root), 22:L6 (Lower molar crown) 23:L6 (Lower molar root), 24:Gla (Glabella), 25:N' (Soft tissue nasion), 26: Sn (Subnasale), 27: SLs (Labialis superior), 28: Sto (Stomion), 29: Li  (Labial Inferior), 30:SLi (Superior Lower labial) 31:HL (H line), 33:HRP (Horizontal Reference Plane), 34:VRP (Vertical Reference Plane), 35: HL (H line drawing tangent to chin and upper lip), 36:FH (Line from Porion to Orbital), 37:SN (line connecting Sella and Nasion), 38:NA (line connecting Nasion and A point), 39:NB (line connecting Nasion and A point), 40:IMPA (Lower incisor mandibular plane angle)

The study aimed to evaluate the degree of tipping and translation of the upper and lower anterior and posterior teeth. This was achieved by marking three reference points on the incisor outlines, namely the root apex (U1-Apex, L1-Apex), center (U1-center, L1-center), and crown tip (U1-edge, L1-edge). Two reference points were also marked mesially on the first molar outline, namely the crown tip (U6-Crown, L6-Crown) and root (U6-Root, L6-Root). The distance from these reference points to the horizontal reference plane (HRP) and vertical reference plane (VRP) was then measured and soft tissue was assessed using Holdaway analysis (Figure 3).

Statistical analysis

The statistical analysis was performed using IBM SPSS software (version 23.0; IBM Corp., Armonk, NY). The normality of the data was evaluated using the Shapiro-Wilk test. Intra-group comparisons between pre- and post-treatment records were conducted using a paired t-test, while inter-group comparisons were performed using an independent t-test.

## Results

On assessment of normality, the data distribution was parametric. Case records of 17 males and 18 females were included in the study. The mean ANB values for groups 1 and 2 were 3.91° ± 1.35° degrees and 4.16° ± 1.5° degrees, respectively. Table 2 depicts the independent t-test p-values for the intergroup difference between t0 and t1 for all the studied parameters. Except for molar mesial movement in group 1, no significant differences in other assessed parameters were noted.

**Table 2:** Independent t-test for intergroup comparison of measured parameters at T0 and T1 Description of all the cephalometric parameters assessed in this study 1: S (Sella), 2: N (Nasion), 3:po (Porion), 4:or (Orbitale), 5:go (Gonion), 6:me (Menton), 7:pog (Pogonion ), 8: Pn (Soft tissue Nose Tip), 9:pog' (Soft tissue Pogonion), 10:Sm (Sub Mentale), 11:A (A point convexity of maxilla), 12:B (B point in convexity of mandible), 13:Upper 1 (Upper incisor), 14:U1 Apex (Upper 1 Root), 15:U1 Center (Upper 1 center), 16: U1 Edge (Upper 1 Crown tip), 17:L1 Apex (Lower 1 Root), 18:L1 Center (Lower 1 center), 19:L1 Edge (Lower 1 crown tip), 20:U6 (Upper molar crown)  21:U6 (Upper molar Root), 22:L6 (Lower molar crown) 23:L6 (Lower molar root), 24:Gla (Glabella), 25:N' (Soft tissue nasion), 26: Sn (Subnasale), 27: SLs (Labialis superior), 28: Sto (Stomion), 29: Li  (Labial Inferior), 30:SLi (Superior Lower labial) 31:HL (H line), 33:HRP (Horizontal Reference Plane), 34:VRP (Vertical Reference Plane), 35: HL (H line drawing tangent to chin and upper lip), 36:FH (Line from Porion to Orbital), 37:SN (line connecting Sella and Nasion), 38:NA (line connecting Nasion and A point), 39:NB (line connecting Nasion and A point), 40:IMPA (Lower incisor mandibular plane angle)

PARAMETERS	P-value of inter group difference at T0	P-value of inter group difference at T1
Soft tissue		
Facial angle	0.26	0.73
Ls Curvature	0.55	0.79
A to N' pog'	0.91	0.16
HL angle	0.83	0.48
PRN to HL	0.58	0.22
SLs - HL	0.12	0.86
A - Sn	0.78	0.66
Ls Strain	0.06	0.43
Strain factor	0.06	0.06
Li - HL	0.36	0.25
SLi - HL	0.11	0.29
Chin thickness	0.29	0.93
Nasolabial angle	0.64	0.73
Mentolabial sulcus	0.46	0.79
Dental parameters		
Upper - SN	0.38	0.60
IMPA	0.29	0.54
Interincisal Angle	0.79	0.22
Incisor		
HRP - L1 Center	0.34	0.43
HRP - L1 Apex	0.54	0.48
HRP - L1 Edge	0.16	0.60
HRP - U1 Apex	0.66	0.73
HRP - U1 Center	0.73	0.54
HRP - U1 Edge	0.43	0.54
VRP - L1 Apex	0.73	0.60
VRP - L1 Center	0.73	0.93
VRP - L1 Edge	0.86	0.79
VRP - U1 Apex	0.60	0.93
VRP - U1 Center	0.48	0.79
VRP - U1 Edge	0.54	0.86
Molar		
VRP-U6 Molar Crown	0.77	0.00
VRP-U6 Molar Root	0.42	0.01
VRP-L6 Molar Crown	0.67	0.00
VRP-L6 Molar Root	0.32	0.04
HRP-U6 Molar Crown	0.14	0.47
HRP-U6 Molar Root	0.18	0.16
HRP-L6 Molar Crown	0.13	0.19
HRP-L6 Molar Root	0.14	0.37
Skeletal parameters		
SNA	0.43	0.25
SNB	0.25	0.34
ANB	0.93	0.66

Table 3 presents the mean and SD of the assessed skeletal, dental, and soft tissue parameters for group 1 at T0 and T1. The paired t-test was conducted to determine the significance of the difference between T0 and T1. In group 1, Soft tissue parameters including Ls Curvature, A to N' pog', HL angle, PRN to HL, and SLs-HL showed significant difference between T0 and T1 (p-value < 0.05). Dental parameters - Upper incisor inclination and interincisal angle had significant difference (p-value < 0.05), whereas no significant difference (>0.05) was noted in lower incisor inclination (T0-T1). There was no significant difference within the group in the movement of upper and lower incisors and molars along their long axis with respect to the HRP between T0 and T1 (p > 0.05). This indicates that the incisors were neither extruded nor intruded while retraction. Statistically significant differences were observed between T0-T1 in the linear distances from the Upper and Lower incisor-root apex, center, and incisal edge, indicating controlled tipping of incisor movement. Additionally, significant differences were noted in Molar mesial crown and mesial root movement to the VRP, with a p-value < 0.05, suggesting anchor loss. Skeletal parameters - SNA, SNB, and ANB showed no significant difference (p-value > 0.05).

**Table 3:** Descriptive statistics and paired t-test of various parameters assessed in group 1 Description of all the cephalometric parameters assessed in this study 1: S (Sella), 2: N (Nasion), 3:po (Porion), 4:or (Orbitale), 5:go (Gonion), 6:me (Menton), 7:pog (Pogonion ), 8: Pn (Soft tissue Nose Tip), 9:pog' (Soft tissue Pogonion), 10:Sm (Sub Mentale), 11:A (A point convexity of maxilla), 12:B (B point in convexity of mandible), 13:Upper 1 (Upper incisor), 14:U1 Apex (Upper 1 Root), 15:U1 Center (Upper 1 center), 16: U1 Edge (Upper 1 Crown tip), 17:L1 Apex (Lower 1 Root), 18:L1 Center (Lower 1 center), 19:L1 Edge (Lower 1 crown tip), 20:U6 (Upper molar crown)  21:U6 (Upper molar Root), 22:L6 (Lower molar crown) 23:L6 (Lower molar root), 24:Gla (Glabella), 25:N' (Soft tissue nasion), 26: Sn (Subnasale), 27: SLs (Labialis superior), 28: Sto (Stomion), 29: Li  (Labial Inferior), 30:SLi (Superior Lower labial) 31:HL (H line), 33:HRP (Horizontal Reference Plane), 34:VRP (Vertical Reference Plane), 35: HL (H line drawing tangent to chin and upper lip), 36:FH (Line from Porion to Orbital), 37:SN (line connecting Sella and Nasion), 38:NA (line connecting Nasion and A point), 39:NB (line connecting Nasion and A point), 40:IMPA (Lower incisor mandibular plane angle)

Soft tissue					
NAME	T0 (Mean±SD)	T1 (Mean±SD)	df	T0-T1 p-value	Inference
Facial angle	90.02°±3.94°	89.15°±3.91°	0.87°±0.02°	0.31	-
Ls Curvature	4.14±1.46 mm	2.43±1.27 mm	1.71±0.18 mm	0.00	Fall back of upper lip
A to N' pog'	4.73±1.91 mm	3.21±1.76 mm	1.52±0.14 mm	0.00	Decreased facial convexity
HL angle	18.36°±2.57°	15.57°±3.11°	2.79°±-0.54°	0.01	Soft tissue Profile Improved
PRN to HL	1.63±2.11 mm	5.33±2.24 mm	-3.7±-0.12 mm	0.00	Nose tip more prominent
SLs - HL	-5.58±1.34 mm	-4.22±1.23 mm	1.36±0.10 mm	0.00	Upper sulcus depth is reduced
A - Sn	13.10±1.82 mm	12.80±2.13 mm	0.3±-0.31 mm	0.41	-
Ls Strain	11.17±1.40 mm	11.13±1.52 mm	0.04±-0.11 mm	1.0	-
Strain factor	1.96±1.19 mm	1.69±1.09 mm	0.27±0.09 mm	0.68	-
Li - HL	2.33±2.43 mm	1.54±2.32 mm	0.79±0.10 mm	0.26	-
SLi - HL	-4.30±1.73 mm	-3.99±1.83 mm	0.31±-0.10 mm	0.41	-
Chin thickness	11.27±1.11 mm	10.66±1.97 mm	0.61±-0.86 mm	0.12	-
Nasolabial angle	94.02±12.41 mm	102.18±12.23 mm	-8.16±0.18 mm	0.01	More obtuse
Mentolabial sulcus	-5.6±0.96 mm	-4.95±1.012 mm	0.65±-0.04 mm	0.09	-
Dental parameters					
Upper 1 - SN	119.95°±4.72°	106.63°±8.18°	13.32°±-3.45°	0.00	Upper anterior Inclination reduced
IMPA	105.76°±6.53°	97.48°±6.63°	8.28°±-0.10°	0.38	Lower anterior inclination did not improved
Interincisal Angle	106.13°±6.64°	121.98°±10.27°	-15.85°±-3.63°	0.00	Incisor inclination reduced
INCISORS:					
HRP - L1 Center	74.01± 4.31 mm	73.55±4.24 mm	1.46±-0.11 mm	0.30	Extrusion
HRP - L1 Apex	80.99±4.57 mm	78.10±4.83 mm	2.09±-0.26 mm	0.37	Extrusion
HRP - L1 Edge	65.12±4.26 mm	64.50±3.81 mm	1.62±0.45 mm	0.24	Extrusion
HRP - U1 Apex	47.40±3.03 mm	45.83±3.74 mm	1.57±-0.70 mm	0.17	Intrusion
HRP - U1 Center	58.18±3.47 mm	55.98±3.89 mm	2.2±-0.41 mm	0.26	Intrusion
HRP - U1 Edge	68.89±4.30 mm	66.08±4.16 mm	2.81±0.13 mm	0.31	Intrusion
VRP - L1 Apex	58.34±3.90 mm	54.78±4.48mm	3.56±-0.58 mm	0.01	Controlled tipping
VRP - L1 Center	65.16±4.09 mm	60.25±4.78 mm	4.87±-0.68 mm	0.01	Controlled tipping
VRP - L1 Edge	71.96±4.66 mm	65.69±5.24 mm	6.27±-0.57 mm	0.00	Controlled tipping
VRP - U1 Apex	63.81±3.86 mm	59.62±3.21 mm	4.19±0.64 mm	0.00	Controlled tipping
VRP - U1 Center	71.35±3.62 mm	64.39±4.28 mm	6.96±-0.66 mm	0.00	Controlled tipping
VRP - U1 Edge	78.9±3.77 mm	69.19±5.72 mm	9.71±-1.94 mm	0.00	Controlled tipping
Molars:					
VRP-U6 Molar Crown	49.15±4.08 mm	51.25±4.55 mm	-2.10±0.50 mm	0.00	Anchor loss
VRP-U6 Molar Root	47.65±3.47 mm	49.77±3.93 mm	-2.12±0.66 mm	0.00	Anchor loss
VRP-L6 Molar Crown	50.92±4.31 mm	52.67±4.52 mm	-1.75±0.38 mm	0.00	Anchor loss
VRP-L6 Molar Root	47.82±3.98 mm	48.97±5.40 mm	-1.15±1.70 mm	0.03	Anchor loss
HRP-U6 Molar Crown	70.02±5.48 mm	68.05±5.79 mm	1.97±0.67 mm	0.11	-
HRP-U6 Molar Root	53.22±5.21 mm	49.25±6.15 mm	3.97±2.95 mm	0.07	-
HRP-L6 Molar Crown	71.12±5.22 mm	68.85±5.97 mm	2.27±1.10 mm	0.09	-
HRP-L6 Molar Root	88.92±5.15 mm	86.62±5.66 mm	2.30±1.23 mm	0.12	-
Skeletal parameters					
SNA	83.44°±2.25°	82.82°±2.68°	0.62°±1.22°	0.16	-
SNB	79.52°±1.99°	80.01°±2.20°	-0.48°±1.10°	0.25	-
ANB	3.91°±1.35°	2.81°±1.37°	1.10°±0.67°	0.06	-

Table 4 depicts the Mean and SD of all assessed parameters (skeletal, dental and soft tissue) in group 2 (T0 and T1) and paired ‘t’ test p-values for the significance of the difference between T0-T1. In group 2 (mini-implant-aided anchorage) Soft tissue parameters - Facial angle, Ls Curvature, A to N' pog', HL angle, PRN to HL, SLs-HL and upper lip strain had significant difference between T0 and T1 (p-value < 0.05). Dental parameters: the linear distance from the incisal edge, centre and root apex of upper and lower incisors to VRP showed a significant difference between T0-T1 (p-value < 0.05), whereas no significant difference (T0-T1) was noted for linear distance from the incisal edge, center and root apex of upper and lower incisors to the HRP (p-value > 0.05).

There was no significant difference (p-value < 0.05) in the linear distance from the mesial crown and root apex of upper and lower molars to VRP and HRP between T0-T1. Skeletal parameter SNA showed a significant difference (p-value < 0.05) whereas no significant difference (p-value > 0.05) was noted in SNB and ANB (Table 4).

**Table 4:** Descriptive statistics and paired t-test of parameters assessed in group 2 Description of all the cephalometric parameters assessed in this study 1: S (Sella), 2: N (Nasion), 3:po (Porion), 4:or (Orbitale), 5:go (Gonion), 6:me (Menton), 7:pog (Pogonion ), 8: Pn (Soft tissue Nose Tip), 9:pog' (Soft tissue Pogonion), 10:Sm (Sub Mentale), 11:A (A point convexity of maxilla), 12:B (B point in convexity of mandible), 13:Upper 1 (Upper incisor), 14:U1 Apex (Upper 1 Root), 15:U1 Center (Upper 1 center), 16: U1 Edge (Upper 1 Crown tip), 17:L1 Apex (Lower 1 Root), 18:L1 Center (Lower 1 center), 19:L1 Edge (Lower 1 crown tip), 20:U6 (Upper molar crown)  21:U6 (Upper molar Root), 22:L6 (Lower molar crown) 23:L6 (Lower molar root), 24:Gla (Glabella), 25:N' (Soft tissue nasion), 26: Sn (Subnasale), 27: SLs (Labialis superior), 28: Sto (Stomion), 29: Li  (Labial Inferior), 30:SLi (Superior Lower labial) 31:HL (H line), 33:HRP (Horizontal Reference Plane), 34:VRP (Vertical Reference Plane), 35: HL (H line drawing tangent to chin and upper lip), 36:FH (Line from Porion to Orbital), 37:SN (line connecting Sella and Nasion), 38:NA (line connecting Nasion and A point), 39:NB (line connecting Nasion and A point), 40:IMPA (Lower incisor mandibular plane angle)

Soft tissue					
NAME	T0 (Mean±SD)	T1 (Mean±SD)	df	T0-T1 p-value	Inference
Facial angle	88.15°±3.48°	90.13°±4.36°	-1.98°±-0.88°	0.02	Improved from class 2 to class 1
Upper lip Curvature	4.3±1.08 mm	2.75±1.62 mm	1.55±-0.54 mm	0.02	Fall back of upper lip curvature
A to N 'pog'	5.16±2.70 mm	5.16±2.15 mm	0±0.6 mm	0.95	-
HL angle	18.41°±3.84°	16.05°±3.27°	2.36°±0.56°	0.02	Soft tissue Profile Improved
PRN to HL	1.08±3.43 mm	4.40±3.01 mm	-3.32±0.41 mm	0.00	Nose tip becomes more prominent
SLs - HL	-6.34±1.33 mm	-4.65±1.67 mm	1.69±-0.34 mm	0.00	Upper sulcus is reduced
A - Sn	12.91±2.10 mm	12.23±2.20 mm	0.68±-0.09 mm	0.37	-
Ls Strain	9.1±1.91 mm	12.31±2.42 mm	-3.21±-0.50 mm	0.01	Improved upper lip strain
Strain factor	3.83±1.57 mm	-0.07±2.14 mm	3.76±-0.56 mm	0.00	Reduced strain factor in upper lip
Li - HL	3.60±1.78 mm	2.73±1.43 mm	0.87±0.35 mm	0.12	-
SLi - HL	-2.83±1.54 mm	-3.1±1.30 mm	0.27±0.24 mm	0.95	-
Chin thickness	10.62±1.82 mm	11.2±2.93 mm	-0.58±-1.11 mm	0.37	-
Nasolabial angle	94.16±9.42 mm	105.37±8.85 mm	-11.21±0.56 mm	0.01	More obtuse
Mentolabial sulcus	-4.92±1.28 mm	-4.71±1.06 mm	0.21±0.22 mm	0.76	-
Dental parameters					
Upper - SN	116.65°±9.68°	102.52°±8.93°	14.13°±0.74°	0.01	Average inclination of upper
IMPA	107.00°±6.48°	96.77°±9.59°	10.23°±-3.11°	0.01	Average inclination of lower
Interincisal Angle/	103.61°±10.89°	129.56°± 12.29°	-25.96°±-1.39°	0.00	Reduced proclination of both upper and lower
INCISORS:					
HRP - L1 Center	75.80±6.63 mm	73.67± 7.43 mm	2.13±-0.80 mm	0.31	Extrusion
HRP - L1 Apex	84.10±6.76 mm	82.05±7.62 mm	2.05±-0.86 mm	0.37	Extrusion
HRP - L1 Edge	67.48±6.63 mm	65.23±7.31 mm	2.25±-0.68 mm	0.23	Extrusion
HRP - U1 Apex	47.63±4.67 mm	44.31±5.15 mm	3.32±-0.48 mm	0.13	Intrusion
HRP - U1 Center	59.15±5.45 mm	55.90±6.43 mm	3.25±-0.98 mm	0.21	Intrusion
HRP - U1 Edge	70.48±6.39 mm	67.48±7.76 mm	3.00±-1.37 mm	0.26	Intrusion
VRP - L1 Apex	55.54±8.24 mm	52.84±8.87 mm	2.7±-0.623 mm	0.06	Controlled tipping
VRP - L1 Center	63.35±7.91 mm	58.35±9.05 mm	5.00±-1.14 mm	0.01	Controlled tipping
VRP - L1 Edge	71.15±7.87 mm	63.85±9.59 mm	7.3±-1.71 mm	0.00	Controlled tipping
VRP - U1 Apex	61.97±6.86 mm	58.70±7.07 mm	3.27±-0.20 mm	0.02	Controlled tipping
VRP - U1 Center	69.64±7.42 mm	62.54±8.41 mm	7.1±-0.98 mm	0.00	Controlled tipping
VRP - U1 Edge	77.28±8.50 mm	70.37±9.89 mm	7.91±-1.38 mm	0.00	Controlled tipping
Molars:					
VRP-U6 Molar Crown	42.17±3.35 mm	42.45±3.72 mm	-0.28±-0.02 mm	0.47	-
VRP-U6 Molar Root	43.05±4.26 mm	42.85±4.37 mm	0.20±-0.11 mm	0.13	-
VRP-L6 Molar Crown	42.55±3.38 mm	42.90±3.49 mm	-0.35±-0.11 mm	0.16	-
VRP-L6 Molar Root	34.42±5.95 mm	34.72±6.16 mm	-0.30±-0.21 mm	0.25	-
HRP-U6 Molar Crown	64.32±1.47 mm	63.15±3.18 mm	1.17±-1.71 mm	0.59	-
HRP-U6 Molar Root	46.15±1.63 mm	45.27±1.97 mm	0.88±-0.34 mm	0.37	-
HRP-L6 Molar Crown	66.20±1.29 mm	66.30±1.64 mm	-0.10±-0.35 mm	0.76	-
HRP-L6 Molar Root	83.82±2.56 mm	83.35±2.89 mm	0.47±-0.33 mm	0.29	-
Skeletal parameters					
SNA	83.15°±3.72°	81.93°±2.73°	1.22°±0.99°	0.01	Decreased
SNB	77.01°±2.29°	76.85°±2.77°	0.15°±0.52°	0.62	-
ANB	4.16°±1.55°	3.47°±2.08°	0.68°±-0.53°	0.05	-

## Discussion

This study utilized two distinct force vectors for anterior teeth retraction and compared the resulting dentoalveolar and soft tissue changes in both sagittal and vertical planes. This study examined two force vectors: one using conventional anchorage parallel to the occlusal plane, and the other using mini-implant-aided anchorage at an angulation of 15-20 degrees from the occlusal plane. This study found no significant difference in the role of force vectors studied for dentoalveolar and soft tissue changes in the sagittal and vertical planes.

Patients with bidental protrusion can expect favorable changes in their facial profile when extraction space is utilized for anterior teeth retraction. In the past, anchor reinforcement was done using trans palatal arches and the Nance palatal button [12]. However, these intraoral appliances could not completely prevent the mesialization of the molars [13,14]. In an attempt to provide absolute anchorage for tooth movement, the skeletal anchorage was therefore developed. TADs like micro or mini-screws have become popular. Previous trials by Liu et al. and Upadhyay et al. employed mini screws to retract six anterior teeth [15,16]. Successful use of these devices can lead to early changes in the facial profile and improved patient cooperation [17]. All subjects in this study underwent treatment that involved the extraction of their four first premolars. After initial leveling and aligning of all teeth, a 0.019x0.025"-inch SS wire was used for retraction.

Chetan et al. conducted a finite element method (FEM) investigation to assess the feasibility of modifying the vertical levels of force application in the posterior region to control the sagittal and vertical movement of maxillary anterior teeth during retraction. The results showed that anterior teeth tipped lingually in the sagittal plane from all points of force application, whereas in the vertical plane, extrusion was seen when retracted from the molar hook, and intrusion was seen when retracted with implants. The retraction component of force decreases by about 1% with every 1 mm of implant apical displacement, whereas the intrusion component of force increases by around 0.3% [11]. Prior studies have reported on comparative changes in incisor angular and bodily positions whereas in the current study, we assessed the incisor movement in sagittal and vertical planes with reference to three points (incisal edge, middle, and root apex). The study observed controlled tipping in patients who received treatment with both conventional and TAD-assisted anchorage, as well as in those who received conventional anchorage. This was due to an accentuated reverse curve of Spee in the upper and lower archwires, which resulted in an intrusive and uprighting force on the anterior teeth. Also, it was reported by Ribero that while using Niti coil springs the tipping and extrusion of incisors can be avoided by giving an exaggerated curve of Spee [18]. No intergroup differences were observed in the distal movement of the incisor in relation to VRP and intrusive movement in relation to HRP upon analyzing the post-treatment records. Molars: Maxillary molars exhibited significant anteroposterior tipping differences on average. Conventional anchorage resulted in greater crown and root movement compared to mini-plant anchorage. In previous studies, molar mesial movement of 1.6 to 4mm during en masse retraction of incisors was reported. Molar anchor loss of 1 to 2 mm is clinically acceptable whereas higher amounts can be detrimental to the overall efficiency of the treatment, especially when anchorage demand is critical. Mini implants are more appropriate for patients who need high or maximum anchorage, particularly for vertical growth patterns [19].

Previous research has examined the impact of removing four premolars and subsequently moving the front teeth backward on soft tissue alterations, particularly in the lips, using traditional anchorage methods [20]. Some studies have reported soft tissue changes with TAD-assisted anchorage [21] but no studies have reported on soft tissue changes with different force vectors for distalizing the anterior segment. According to Baik et al., the soft tissue changes in cases treated with extraction of the four premolars followed by en masse retraction of anterior teeth showed sufficient fallback of soft tissue points A and B [22]. This study analyzed two force vectors from different anchorage systems (mini-implant aided and conventional anchorage) in subjects with bi-dental proclination who underwent the first four premolar extractions. Soft tissue parameters (Ls Curvature, A to N' pog', HL angle, PRN to HL, SLs-HL) were measured using sagittal and vertical reference planes. Significant differences between t0 and t1 were observed in both groups. Significant intergroup differences were noted for a facial angle which increased in subjects treated with the mini-implant anchorage group. The facial angle and the position of the upper lip are considered essential factors in assessing soft tissue profiles because they affect the esthetic features of skeletal class II patients.

The upper and lower lips influence the aesthetics of the lower third of the face and the upper lip attracts the greatest attention [23]. Superior sulcus depth was negatively related to Class II indicating superior sulcus depth is the only ULC that might be significantly corrected by the intervention of skeletal growth [24,25]. Comparing the mini-implant-aided group and conventional group, the changes in SLs - HL (upper sulcus depth is measured from the H line) were 1.69±-0.341 mm and 1.36±0.107 mm, respectively, the upper sulcus was reduced more in the mini-implant-aided group than compared to the Conventional anchorage group. However, the changes in Ls Strain (Horizontally from the maxillary central incisor to the vermilion border of the upper lip) were -3.21±-0.508 mm in mini-implant aided and 0.04±-0.119 mm in the conventional group. The upper lip strain is improved more in the mini-implant-aided group than in the conventional group with no significant difference.

The results of the current study may differ from those of earlier studies as a consequence of many factors. There were only adult patients in the sample. Patients who received treatment before the age of 13 had a considerably higher mesial displacement of the maxillary first molars (by a mean of 1.2 mm) than did patients who began treatment at subsequent ages, according to research by Xu et al. [26]. Two force vectors on different anchorage devices were used during space closure in the current study. Previous studies [26,27] demonstrated only the retraction rate combined with mini implants. The friction may have interfered with, and modified movement rates and tooth rotation may have occurred [28].

Limitations

The generalization of these results to clinical settings is subject to certain limitations, such as the influence of variables such as age, gender, dentofacial morphology, and ethnicity on the accurate prediction of soft tissue changes following tooth movement. As the retention period was not reported, there might have been significant post-treatment alterations. These considerations should be taken into account in future research.

## Conclusions

The outcomes of our research underscore a noteworthy advancement in orthodontic practice. Specifically, our study illuminates that the application of carefully examined force vectors for distal incisor movement subsequent to premolar extractions yields multifaceted benefits. Most notably, this approach brings about a distinct enhancement in lip procumbency and incisor inclinations, contributing to an overall improvement in facial aesthetics. A compelling aspect to emphasize is that these favorable effects are achieved without engendering any substantial alterations in the underlying skeletal framework, which holds considerable significance for the planning and execution of orthodontic interventions. It is also intriguing to note that in contrast, individuals subjected to conventional anchorage experience mesial movement of molars, further highlighting the nuanced interplay between force dynamics and dental positioning. This comprehensive understanding derived from our study advances our comprehension of dental biomechanics and holds implications for the development of refined strategies for managing malocclusions and optimizing facial harmony.
